# Supplementary material for: Sushi domain-containing protein 4 controls synaptic plasticity and motor learning
Source: eLife. 2021 Mar 4;10:e65712. doi: 10.7554/eLife.65712 (PMC7972451; doi:10.7554/eLife.65712)
Supplement: Supplementary file 1. [file elife-65712-supp1.docx]

**­­­**

**Supplementary Table 1. Behavioral characterization of *Susd4* KO mice.** From three month-old *Susd4* knockout (KO) and *wild type* (WT) littermates. Mean ± s.e.m. or percentage of mice (Physical Characteristics: WT n=10 and KO n=12 mice; Sensorimotor Reflexes and Motor responses: WT n=24 and KO n=24 mice).

|  | WT | | *Susd4* KO | |
| --- | --- | --- | --- | --- |
| *Physical Characteristics* |  |  |  |  |
| Weight (g) | 24,13 | ± 1.20 | 24,72 | ± 1.33 |
| Whiskers (% with) | 80 | % | 83,3 | % |
| Palpebral Closure (% with) | 0 | % | 0 | % |
| Piloerection (% with) | 20 | % | 25 | % |
| *Sensorimotor Reflexes* |  |  |  |  |
| *(% subjects displaying "normal response")* |  |  |  |  |
| Cage movement | 100 | % | 100 | % |
| Whisker response | 100 | % | 100 | % |
| Eye Blink | 100 | % | 100 | % |
| Ear Twitch | 100 | % | 100 | % |
| *Motor Responses* |  |  |  |  |
| Open Field Locomotion |  |  |  |  |
| Improvement (number) | 22.83 | ± 2,89 | 19,42 | ± 2,03 |
| Distance (cm) | 2764 | ± 235.0 | 2301 | ± 158,5 |
| Speed (cm/s) | 13.32 | ± 0,42 | 13,06 | ± 0,30 |
| Time on Center (%) | 13.30 | ± 1,33 | 11,18 | ± 1,26 |
